# Study of β-Ga_2_O_3_ Ceramics Synthesized under Powerful Electron Beam

**DOI:** 10.3390/ma16216997

**Published:** 2023-11-01

**Authors:** Abay B. Usseinov, Zhakyp T. Karipbayev, Juris Purans, Askhat B. Kakimov, Aizat Bakytkyzy, Amangeldy M. Zhunusbekov, Temirgali A. Koketai, Artem L. Kozlovskyi, Yana Suchikova, Anatoli I. Popov

**Affiliations:** 1 Faculty of Physics and Technical Sciences, L.N. Gumilyov Eurasian National University, Astana 010008, Kazakhstan; usseinov_ab@enu.kz (A.B.U.); kakimov_ab_2@enu.kz (A.B.K.); kukenova_ab_1@enu.kz (A.B.); zhunusbekov_am@enu.kz (A.M.Z.); kozlovskiy.a@inp.kz (A.L.K.); 2 Institute of Solid State Physics, University of Latvia, 8 Kengaraga Str., Lv-1063 Riga, Latvia; purans@cfi.lu.lv; 3 Department of Physics and Nanotechnology, Karaganda Buketov University, Karaganda 100028, Kazakhstan; katkargu@mail.ru; 4 The Department of Physics and Methods of Teaching Physics, Berdyansk State Pedagogical University, 71100 Berdyansk, Ukraine; yanasuchikova@gmail.com

**Keywords:** synthesis, power electronic flux, β-Ga_2_O_3_ ceramics, photoluminescence, annealing, point defects

## Abstract

The synthesis of β-Ga_2_O_3_ ceramic was achieved using high-energy electron beams for the first time. The irradiation of gallium oxide powder in a copper crucible using a 1.4 MeV electron beam resulted in a monolithic ceramic structure, eliminating powder particles and imperfections. The synthesized β-Ga_2_O_3_ ceramic exhibited a close-to-ideal composition of O/Ga in a 3:2 ratio. X-ray diffraction analysis confirmed a monoclinic structure (space group C2/m) that matched the reference diagram before and after annealing. Photoluminescence spectra revealed multiple luminescence peaks at blue (~2.7 eV) and UV (3.3, 3.4, 3.8 eV) wavelengths for the synthesized ceramic and commercial crystals. Raman spectroscopy confirmed the bonding modes in the synthesized ceramic. The electron beam-assisted method offers a rapid and cost-effective approach for β-Ga_2_O_3_ ceramic production without requiring additional equipment or complex manipulations. This method holds promise for fabricating refractory ceramics with high melting points, both doped and undoped.

## 1. Introduction

Gallium oxide (Ga_2_O_3_) is a fascinating compound with a wide bandgap, high breakdown electric field, and remarkable thermal and chemical stability, making it an excellent candidate for high-power electronic devices, ultraviolet (UV) light-emitting diodes, and gas sensors. In addition, Ga_2_O_3_ demonstrates the immense potential for application in advanced scintillators and phosphors owing to its wide bandgap, high thermal stability, and resistance to radiation damage. The luminescence properties of Ga_2_O_3_ are further enhanced via doping with rare-earth ions or other luminescent centers.

### 1.1. Main Properties and Application

Ga_2_O_3_ has five stable phases: α, β, γ, δ, and ε [1,2,3,4]. Among them, the β-phase is the most stable and extensively studied due to its extraordinary electrical and optical properties. β-Ga_2_O_3_ has a wide bandgap (4.9 eV) [5] and high breakdown field (~8 MV/cm) [6,7,8], giving it the potential to outperform the traditional silicon carbide (SiC) and gallium nitride (GaN) used in power devices.

β-Ga_2_O_3_ has shown promising scintillation characteristics [9,10,11,12]. The unique properties of β-Ga_2_O_3_ make it an intriguing option for these and similar applications. Its wide bandgap reduces self-absorption, enhancing light yield, while its excellent thermal stability and radiation hardness allow for application in harsh environments. Further, β-Ga_2_O_3_ has a high atomic number and density which enhance its photoelectric absorption and Compton scattering properties, contributing to a more efficient scintillation process.

### 1.2. Rare-Earth Doping β-Ga_2_O_3_

Doping β-Ga_2_O_3_ with rare-earth ions like Eu^3+^, Ce^3+^, and other luminescent centers (e.g., Tb^3+^, Tm^3+^) has been studied extensively to enhance the scintillation efficiency [12,13,14]. The doping process helps create energy levels within the bandgap, facilitating the scintillation process. However, the main challenge with β-Ga_2_O_3_ scintillators is reducing the non-radiative transitions, which impact the scintillation efficiency. Research into achieving higher crystal quality and efficient doping methods is ongoing.

As it was reported, β-Ga_2_O_3_-based phosphors have shown significant potential in the field of optoelectronics, especially in white light-emitting diodes (LEDs) [15]. The key to the successful application of Ga_2_O_3_ phosphors lies in the choice of the appropriate activators. Rare-earth ions such as Eu^3+^ (red emission), Tb^3+^ (green emission), and Ce^3+^ (blue emission) have been extensively studied as activators [16,17,18]. Further, Ga_2_O_3_ phosphors exhibit high thermal stability, which is a crucial factor for their operation in devices like LEDs. However, the efficiency of these phosphors is still less compared to established phosphor materials, and research is ongoing to improve their luminescence characteristics.

Overall, gallium oxide demonstrates significant potential as a scintillator and phosphor material, with its properties being enhanced through the process of doping. However, additional research is required to overcome the challenges of non-radiative transitions in scintillators and low efficiency in phosphors. The continuous improvement in synthesis techniques and a deeper understanding of material properties are expected to unlock the full potential of Ga_2_O_3_ in these applications.

### 1.3. Methods of Synthesis

Multiple techniques, such as the casting method [19], the edge-defined film-fed growth (EFG) method [20], Czochralski method [21,22], Bridgman method [23], pulsed laser deposition [24], and hydrothermal method [25] can be employed for the growth of bulk β-Ga_2_O_3_ crystals. The EFG method is preferred due to its ability to produce large-sized, high-quality β-Ga_2_O_3_ crystals [26]. Nevertheless, the quality of the grown crystal depends on factors such as temperature, pulling speed, and precursor material.

Epitaxial growth methods, such as Metal-Organic Chemical Vapor Deposition (MOCVD) [27] and Molecular Beam Epitaxy (MBE) [28] are used to grow thin films of Ga_2_O_3_ on suitable substrates like sapphire, silicon carbide, or Ga_2_O_3_ itself. The epitaxial growth methods have been successful in growing high-quality β-Ga_2_O_3_ films [29] but the process requires stringent control over parameters to avoid defects and maintain crystallographic orientation.

The hydrothermal method is widely used to synthesize Ga_2_O_3_ nanocrystals and nanowires due to its low temperature, simple apparatus, and tunable morphology. The reaction is carried out in a high-pressure autoclave where the precursors react in the presence of mineralizers. However, the hydrothermal method is time-consuming and requires subsequent annealing to remove impurities and defects.

Ceramic Ga_2_O_3_ can be synthesized via solid-state reaction methods [30], sol-gel methods [31], or spark plasma sintering [32]. These methods allow the formation of complex shapes and sizes but often lack the level of crystallographic order and purity needed for electronic applications.

In summary, while the synthesis of Ga_2_O_3_ crystals and ceramics has seen significant advancements, challenges still exist in terms of controlling purity, morphology, and crystallographic orientation. Future work should focus on optimizing growth conditions and developing novel synthesis methods to produce high-quality, large-size Ga_2_O_3_ crystals and ceramics for broad-scale industrial applications.

### 1.4. Electron-Beam Assisted Synthesis

The advent of electron-beam assisted synthesis (e-beam) technology has provided new avenues for ceramic synthesis, with the capability to fabricate complex and novel ceramic structures. The synthesis of ceramics using traditional methods often involves high-temperature processing, which can lead to grain growth, phase segregation, and structural inhomogeneities. E-beam technology emerges as a promising alternative, offering precise control over fabrication parameters, localized high-energy irradiation, and high production rates. In e-beam assisted synthesis, a focused beam of high-energy electrons interacts with the precursor material. The energy transferred initiates physical or chemical reactions, leading to the formation of ceramic structures. E-beam assisted synthesis presents several advantages over conventional methods: the process can be performed rapidly due to the high-energy nature of the e-beam; the method is compatible with various precursors and substrates, enabling the synthesis of a wide range of ceramic compositions and structures.

In addition to the previously discussed techniques, recent studies have unveiled impressive results on the fabrication of refractory ceramics, specifically magnesium fluoride (MgF_2_) and yttrium-aluminum garnet (YAG) ceramics, utilizing a powerful electron beam [33,34,35]. Consequently, there is a heightened interest in the development and refinement of this newly proposed synthesis method for refractory materials employing a powerful electron beam.

## 2. Materials and Methods

**Figure 1:**
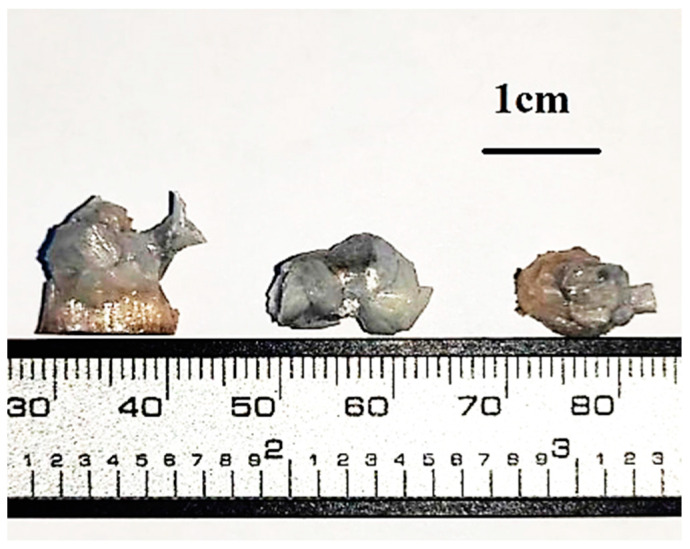
β-Ga_2_O_3_ ceramics synthesized under a powerful electron beam.

β-Ga_2_O_3_ crystals were synthesized via irradiation of the initial β-Ga_2_O_3_ powder with 99.999% purity using a powerful electron beam generated by an ELV-6M electron accelerator [36]. The powder was placed in a copper crucible with recess of 5 mm. As a result of irradiation, the powder sample fused, forming small irregularly shaped ceramics with linear dimensions of about 1 cm (Figure 1). The energy of accelerated electrons was 1.4 MeV. The power of the accelerated electron flux was ~25 kW/cm^2^, and the total irradiation time was 36 s. The resulting ceramic pieces were cooled to room temperature, divided into several parts, and examined. After structural and spectral investigations, the test sample was then annealed in air for 6 h at 1000 °C and the properties of the annealed sample were re-examined.

**Figure 2:**
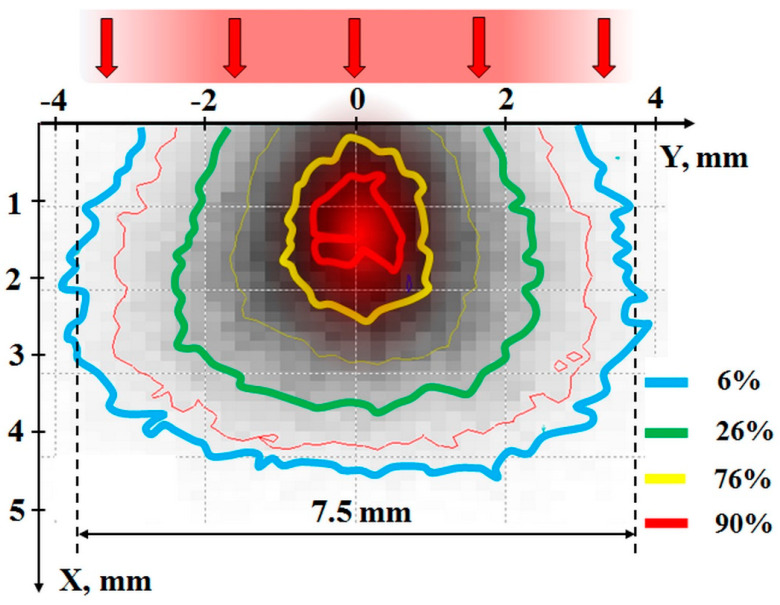
Absorbed dose distribution in the mixture when exposed to electrons at 1.4 MeV energy.

The study of the atomic structure of the resulting ceramics was carried out on a D8 ADVANCE ECO diffractometer. Diffractograms were built in the range of angles 2θ: 20–110° with a step of 0.02° (Figure 2). The quantitative ratio of the phases was determined in the TOPAS 4.2 program. The surface state of the synthesized ceramic samples was studied using a Hitachi TM3030 scanning electron microscope (Hitachi High-Technologies Corporation, Tokyo, Japan) with a Bruker XFlash MIN SVE energy dispersive analysis system (energy-dispersive spectroscopy, EDS) (Bruker, Billerica, MA, USA) at an accelerating voltage of 15 kV, which was used to analyze the composition of β-Ga_2_O_3_. In order to analyze the electronic and optical properties of the resulting ceramics, we measured the excitation and PL emission spectra of the obtained samples in the wavelength range from 200 to 800 nm on a CM-2203 spectrofluorimeter (Solar, Minsk, Belarus) at room temperature (RT). Raman spectra were recorded using Horiba Jobin–Yvon LabRam HR800 (Horiba Jobin Yvon, Longjumeau, France). To evaluate the obtained data, we compared the obtained results with the same results that have been received on commercial unintentional doped (UID) crystals of β-Ga_2_O_3_ with orientation (−201) from Tamura Corp. (Tokyo, Japan) [37].

## 3. Results

### 3.1. Assessment and Analysis of Electron Energy Loss Distribution in a Substance upon Irradiation

In order to study the development of the melting process under the influence of an electron beam, we undertook a comprehensive numerical simulation to explore electron energy losses when passing through Ga_2_O_3_ powder using the advanced Casino v2.51 code [38]. This simulation was achieved through the Monte Carlo methods. We set the following parameters: electron energy 1.4 MeV, beam diameter 7.5 mm, and Ga_2_O_3_ powder bulk density 1.5 g/cm^3^. Our exhaustive calculations were based on the passage of a substantial count of 10,000 electrons through the Ga_2_O_3_ powder, and the consequential data are graphically illustrated in Figure 2. In our experimental design, the synthesis was instigated by subjecting a blend of powders to an electron beam.

An in-depth look at the findings depicted in Figure 2 reveals a fascinating phenomenon. As the electron beam, with a confined cross-section, traverses the substance, there is a distinct redistribution of energy losses. Some energy gets channeled to the material beyond the beam’s confining aperture, while a fraction is reallocated towards the beam’s epicenter during its trajectory. This reallocation results in the energy loss density along the beam’s center surpassing its peripheral counterpart. There is a noticeable growth in the energy loss of the electron stream up to a specific depth before it starts to recede. The culmination of these dynamics results in the energy losses being predominantly concentrated along the beam axis, especially in regions distanced from the surface. Figure 2 elucidates the zones where energy loss is of equal magnitude, represented in relative units. The segment showcasing the most significant energy losses is distinctively demarcated with solid shading. When examining Ga_2_O_3_ with a bulk density of 1.5 g/cm^3^, prepared for the synthesis process, nearly half of the total energy gets absorbed within a 4.5 mm diameter area in a cross-section perpendicular to the electron’s angle of incidence, ranging from 1.4 to 2.9 mm from the surfaces. Remarkably, the energy loss density at the core is at least quintuple the volume average.

An essential observation to make is the distribution of electrons within our beam, which exhibits a Gaussian pattern. Consequently, the beam’s density along the ingress axis significantly outstrips its peripheral counterpart. This distinctive arrangement implies that, in a tangible scenario, the observed phenomenon of energy absorption concentrated along the beam’s passage axis within the material should be even more accentuated.

Earlier studies have provided a ballpark estimation of the temperature at which the ceramic precursor, commonly referred to as the “charge”, is heated during its formation [39]. This temperature is directly influenced by the intensity of the electron irradiation applied. Specifically, when the charge undergoes exposure to an electron flux with a power density of 20 kW/cm^2^, it results in the material heating up to temperatures surpassing 1500 °C. Such a marked temperature threshold can be attributed to the underlying processes during the synthesis phase. Notably, it is the ionization-related processes of the material that seem to have a preeminent role in dictating this temperature threshold.

The process of ceramic formation is intricately linked to the radiolysis of the originating powder particles. Specifically, it is the intermediate products that result from this radiolysis that play a pivotal role in the eventual development and constitution of ceramics.

### 3.2. XRD Analysis

**Figure 3:**
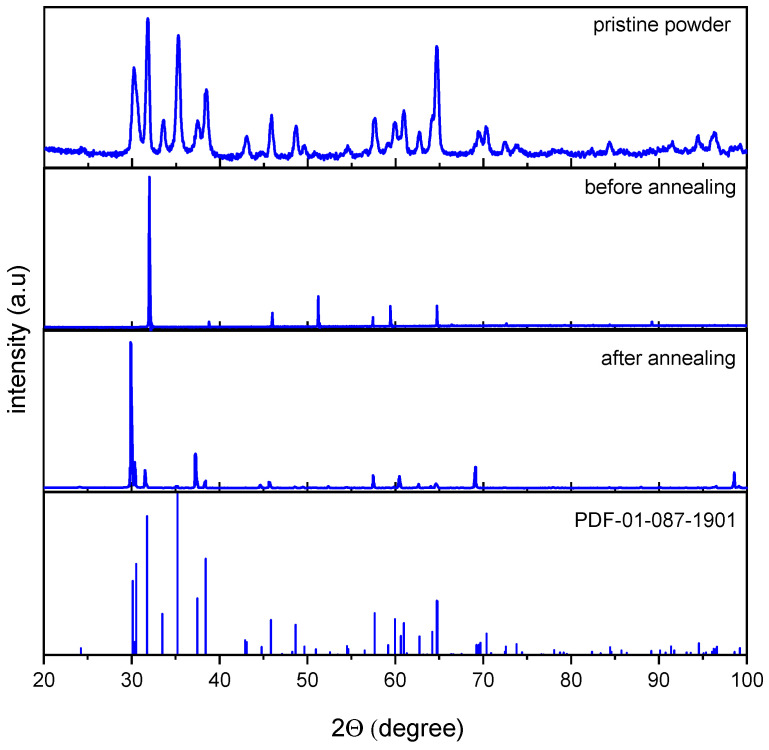
X-ray diffraction patterns of the synthesized β-Ga_2_O_3_ before and after annealing. The standard PDF-01-0871901 [40] was placed at bottom for comparison.

Figure 3 shows the XRD patterns of the synthesized β-Ga_2_O_3_ ceramic before and after annealing in comparison with ICSD pattern (PDF-01-0871901) [40], respectively. The diffraction patterns of the synthesized β-Ga_2_O_3_ ceramics before and after annealing (Figure 3) have a good comparison with the reference diagram, which indicates that the obtained samples correspond to the monoclinic structure of the space group C2/m. It can be assumed that a sharp local increase in temperature during irradiation using a powerful electron beam leads to remains of deformations and distortions of the crystal structure formed during powder sintering. In this case, the most pronounced distortion of the crystal lattice is observed along the *a* and *b* axes, for which the deviation from the reference values is more than 1% (Table 1 and Table 2). Indeed, the observed slight deviation of the crystal lattice parameters from their reference values, as well as the smaller unit cell volume compared to the reference unit cell, indicates distortions of the crystal lattice which can be attributed to the “detonation” nature of the initial powder sintering. Subsequent annealing causes the diffraction peaks to slightly shift towards lower values of the Bragg angle, which indicates an increase in the lattice parameters and unit cell volume, improving the convergence with the reference values, increasing the crystallinity of the lattice, and decreasing the crystallite size. Thus, annealing leads to the removal of residual stresses and distortions in the lattice, preserved as a result of rapid sintering of the β-Ga_2_O_3_ powder (Table 1 and Figure 3). Note that the degree of sample crystallinity has estimated the ratio of the integrated intensity of the reflections to the total X-ray intensity.

### 3.3. Surface Morphology and Elemental Composition

**Figure 4:**
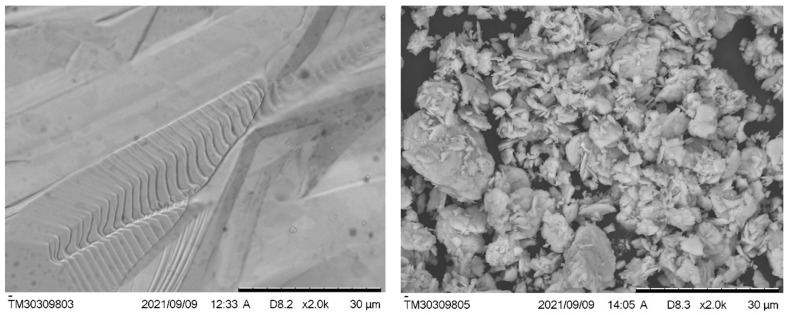
SEM images of synthesized ceramics (**left**) and the initial Ga_2_O_3_ powder (**right**).

The used apparatus made it possible to observe volumetric samples with shadow and volumetric contrast with a resolution of up to 30 nm. Figure 4 shows typical SEM images of the synthesized β-Ga_2_O_3_ ceramic surfaces with an area of ~0.016 mm^2^ (a) and powdered sample (b), magnified by 1000 times. Evidently, the resulting ceramics have a monolithic surface structure, which indicates the complete disappearance of powder particles or any other imperfections with formation of a solid phase. The original gallium oxide powder consists of particles ranging in size from 1 to 15 µm (Figure 4, right).

**Figure 5:**
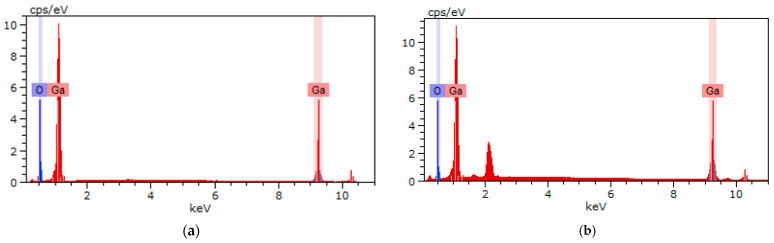
EDS result for sintered β-Ga_2_O_3_ ceramic before (**a**) and after (**b**) annealing.

The obtained β-Ga_2_O_3_ ceramic composition is close to ideal O/Ga ratio of 3/2 (Table 3, Figure 5) [41] and comparable with the earlier obtained composition of β-Ga_2_O_3_ nanowires [42]. It has been found that post-annealing greatly changes the O/Ga ratio, whereby the Ga content decreases significantly and the O content increases. An increase in oxygen concentration is associated with the absorption of oxygen from the air, as well as with a decrease in oxygen vacancies in crystal structure. In addition, the stoichiometry of the initial powder was shown, which is close to the stoichiometry of annealed sample.

### 3.4. Luminescence Spectra of Sintered and Commercial β-Ga_2_O_3_ Ceramic

**Figure 6:**
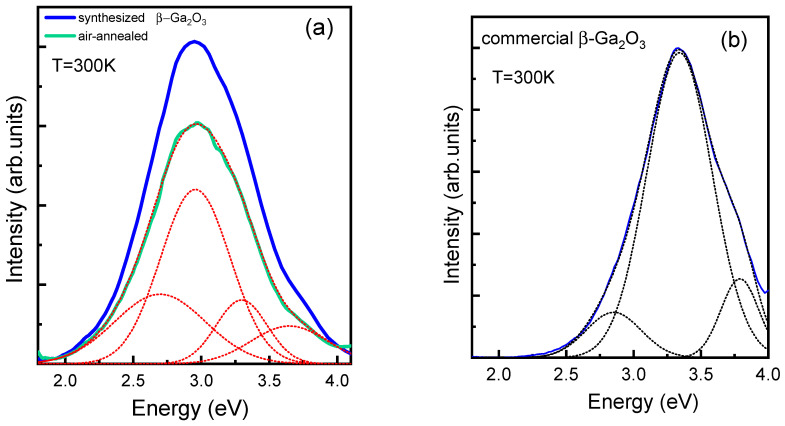
Luminescence spectra of synthesized β-Ga_2_O_3_ ceramic before and after annealing (**a**) and commercial β-Ga_2_O_3_ crystal (**b**). Excitation energy 4.9 eV.

The photoluminescence spectra of the synthesized ceramics and commercial crystals upon excitation with an energy of 4.9 eV are shown in Figure 6a,b. Through Gaussian approximation, we found three components with peaks corresponding to blue (~2.7 eV) and UV (3.3, 3.4, 3.8 eV) luminescence (Figure 6a). It can be seen that the spectra measured for synthesized ceramics and commercial crystals are quite similar. The main difference between spectra is that the position of a luminescence peak of synthesized ceramics exhibits in a lower energy range of around 3 eV (Figure 6a). The latter indicates the presence of distortions and defects in the crystal structure, which cause the deviation of the electronic properties from the electronic properties of commercial crystals. After annealing, the concentration of defects reduces, improving crystal quality and causing a decrease in the luminescence intensity of ~27% (Figure 6a). The downward trend in intensity after annealing is also confirmed in studies of β-Ga_2_O_3_ crystals obtained via edge-defined film-fed growth (EFG) [41], as well as in the study of a variety of YAG:Ce, YAG:Gd, and Ce ceramics [34]. Intensity decreasing can be ascribed to two possible reasons: a decrease in the concentration of oxygen vacancies (V_O_), as well as the capture of electrons involved in luminescence in traps (for instance, in Ga atoms). Also, during annealing, impurities with a low migration barrier, such as hydrogen [43], can leave the crystal. As shown by recent calculations, hydrogen in β-Ga_2_O_3_ is a shallow donor [43,44] and can participate in luminescence processes.

Since the excitation of UV luminescence in β-Ga_2_O_3_ crystals occurs along the edge of the absorption band, and the maximum of this excitation band coincides within the experimental error with the maximum of the photoconductivity excitation band, we believe that the absorption band in which UV luminescence is excited is the band of the interband transition [45,46]. Thus, UV luminescence can be attributed to the recombination of free electrons and self-trapped holes, and not to lattice defects. It is known from the literature that the UV luminescence are independent of the history of the crystal, the presence of impurities, or the growth condition and instead is due to the recombination of excited electrons with self-trapped holes (STHs) [41,44,45,46,47].

Early studies of blue luminescence showed that it is due to charge recombination in donor-acceptor defects [46,47,48,49]. The evidence that defect levels are involved in the blue luminescence is also indicated by the fact that the blue emission can be excited via irradiation with an energy 4.67 eV lower than the band gap 4.9 eV [47]. Furthermore, excitation of blue luminescence was observed in both doped and undoped samples. The latter circumstance indicates the participation of intrinsic donor and acceptor defects in the blue luminescence. O vacancies and interstitial Ga can act as donors, while Ga vacancies and/or di-vacancies (V_Ga_-V_O_) can act as acceptors [47,50]. Our recent hybrid DFT simulations on single-vacancy defects showed that O vacancies are deep donors, while Ga vacancies are deep acceptors [51,52]. Both can be compensated for each other; thus, preferable charge states are 2+ for O vacancies and 3- for Ga vacancies. Next, we have performed a modeling of pair vacancies (di-vacancies) and shown that di-vacancies can be effectively accumulated in a β-Ga_2_O_3_ crystal along with single vacancies due to low formation energy; they may play a role of deep donors at low Fermi energies, and deep acceptors at high Fermi energies [53].

**Figure 7:**
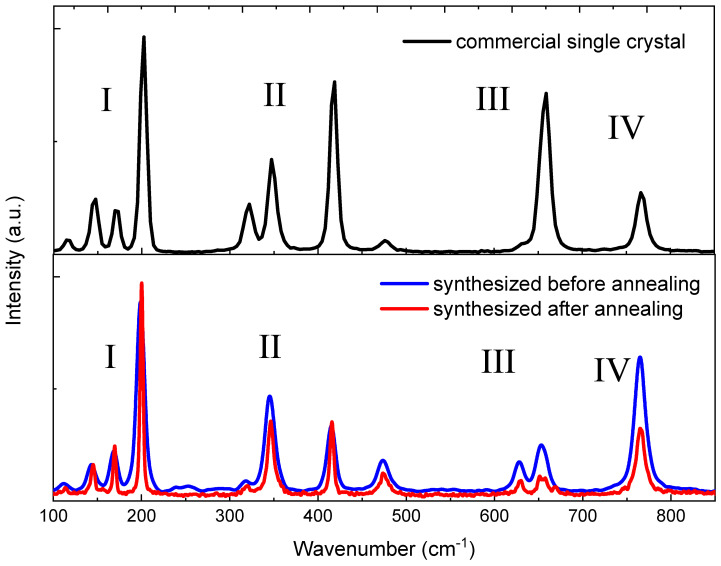
The Raman spectra for commercial single crystal and synthesized Ga_2_O_3_ ceramics before and after annealing.

In order to study the effect of annealing on the valence states of Ga sublattice, we measured the Raman scattering spectra which reflect the vibrations of valence bonds. These spectra recorded for the synthesized β-Ga_2_O_3_ ceramics (before and after annealing) and commercial crystals are shown in Figure 6 and Table 4. Eleven peaks were found for all samples, which are consistent with the data in the literature [41,54,55,56,57] (see Figure 6). As a rule, the low-frequency zone (I) is attributed to the vibration and translation of tetrahedral-octahedral chains, the mid-frequency zone (II) reflects deformations of the GaO_6_ octahedron, and the high-frequency zones (III) and (IV) refer to stretching and bending of the GaO_4_ tetrahedron [57,58]. Some notable conclusions can be drawn by comparing the spectra before and after annealing. Considering that the spectra in lower Figure 7 are normalized at a frequency of 200 cm^−1^, we can conclude that, firstly, after annealing the contribution of peaks three and four drops significantly, and secondly, the peak at 655–660 cm^−1^ shows a doublet structure. Understanding this behavior, although it indicates a structural transformation of the synthesized ceramics, nevertheless requires a more detailed ab initio analysis of the Raman spectra with structural defects (vacancies, pairs of vacancies, etc.), which requires additional dedicated work. Recently, similar work was conducted, for example, for SrTiO_3_ crystal with oxygen vacancies [59,60]. 

Why annealing of Ga_2_O_3_ ceramics can greatly change the concentration of vacancies, as well as their transformation into more complex ones, is not such a difficult question. In fact, it is well known that in all binary oxides (MgO, Al_2_O_2_, ZrO_2_) and complex oxides (MgAl_2_O_4_) in the region of 200–600 °C, this change was observed more than once. 

Before moving on to the conclusions, it should be noted that the methodology discussed in this work certainly needs further improvement. This is especially important when it is necessary to obtain transparent samples, for example, for various optical applications or photo detection [61].

## 4. Conclusions

We have studied the structural, luminescent, and vibronic characteristics of β-Ga_2_O_3_ ceramics synthesized from a powdered sample under the action of a powerful electron beam, in comparison with the same characteristics for commercial crystals used in the production of solar-blind photodetectors and scintillators. The constructed diffraction patterns for the synthesized ceramics and the standard (reference) print PDF-01-0871901 show the similarity of the atomic structure and phase of the synthesized ceramics and commercial samples. Subsequent annealing at 1000 °C has a favorable effect on the structural and composite properties of the resulting ceramics in comparison with commercial samples: annealing leads to the removal of residual stresses and the “leveling” of the monoclinic structure. In addition, annealing leads to an improvement in the Ga/O ratio compared to commercial crystals. SEM studies of the surface showed that the sintered ceramic has a monolithic structure without the formation of aggregates and residual defects; therefore, it can be assumed that, as a result of electron irradiation, the entire initial powder material reacted completely in the formation of a crystalline phase.

The spectral properties of the synthesized ceramics are also comparable to those of commercial crystals. The observed difference between the luminescence spectra of the synthesized ceramics and commercial crystals excited in the fundamental absorption band is due to the presence of residual distortions and defects, which are significantly reduced after subsequent annealing. After annealing, UV luminescence still has a dominant contribution to the total spectrum while blue luminescence is reduced due to the partial annealing of oxygen vacancies.

The electron beam-assisted method can provide faster and cheap production of β-Ga_2_O_3_ crystals without the use of additional equipment or manipulations. The method is effective for the production of doped and undoped refractory ceramics with high melting points. 

## Figures and Tables

**Table 1:** Lattice parameters and crystallinity of β-Ga_2_O_3_ before and after 6 h annealing at 1000 °C in air.

	a (Ǻ)	b (Ǻ)	c (Ǻ)	β (deg)	V (Ǻ3)	Density (g/cm^3^)	Crystallites Size (nm)	Crystallinity (%)
Pristine Ga_2_O_3_ powder	12.17376	3.04365	5.77367	103.291	208.20	5.4874	22.34	76.1
Before annealing Ga_2_O_3_	12.08948	3.00727	5.76147	103.651	203.55	5.6128	141.06	79.9
After annealing Ga_2_O_3_	12.23419	3.04665	5.81359	103.922	210.33	5.4318	34.82	90.5
PDF-01-0871901	12.2140	3.0371	5.7981	103.830	208.846	5.4704		–

**Table 2:** Position of diffraction peaks and crystallographic directions of the powder sample and synthesized β-Ga_2_O_3_ ceramics.

2θ (Degree)				h k l
Reference	Pristine Powder	Before Annealing	After Annealing	
30.36	30.22		29.97	1 1 0
31.76	31.74	31.93	31.56	−2 0 2
33.49	33.57			−1 1 1
35.22	35.22			1 1 1
37.48	37.48		37.27	4 0 1
38.42	38.44	38.76	38.42	−4 0 2
43.10	43.06			−1 1 2
45.84	45.86	45.98	45.69	1 1 2
48.67	48.67		48.55	5 1 0
49.66	49.62		49.48	4 0 2
50.95	50.76	51.2	50.77	−4 0 3
54.51	54.55		54.49	5 1 1
56.53	56.48			−1 1 3
57.66	57.65	57.44	57.46	−3 1 3
59.19	59.08	59.44	59.14	−6 0 3
59.97	59.915	59.6	59.84	1 1 3
60.96	60.96		60.43	0 2 0
62.75	62.69		62.66	7 1 0
64.20	64.21		64	−2 0 4
64.71	64.69	64.73	64.61	−7 1 2
69.49	69.4		69.12	4 2 0
70.39	70.27			0 2 2
72.47	72.35	72.64	73.41	−6 0 4
73.80	73.78			4 2 1
82.38	82.35	82.48		−8 0 4
84.44	84.38	84.41	84.31	−7 1 4
89.13		89.23	90.07	−6 0 5
91.38	91.51		91.31	−8 2 1
93.14	93.06		92.93	8 2 0
94.56	94.46		94.39	−2 2 4
96.23	96.28		96.48	5 1 4

**Table 3:** Elemental analysis of the powder sample and synthesized β-Ga_2_O_3_ ceramics (in at.%).

Atom	Synthesized β-Ga_2_O_3_		Pristine Powder β-Ga_2_O_3_
	Before Annealing	After Annealing	
Ga	39.31	33.17	33.40
O	60.69	66.83	66.60
O/Ga ratio	1.54	2.0	1.99

**Table 4:** Shifts in peak positions and changes in FWHM observed in the Raman spectra of commercial crystal and β-Ga_2_O_3_ ceramics samples before and after annealing (in at.%).

Commercial Single Crystal		Ceramic Sample before Annealing		Ceramic Sample after Annealing		[54]	[55]	[56]	[57]	[54]
ν, cm^−1^	FWHM, cm^−1^	ν, cm^−1^	FWHM, cm^−1^	ν, cm^−1^	FWHM, cm^−1^	Experiment				Theory
						ν, cm^−1^				
115.7	8.1	111.4	0.4	113.9	3.6	114.8	114	113.6	115	118.6
146.3	9.5	143.1	0.4	145.0	5.3	144.8	147	144.7	149	145.6
171.1	8.3	168.7	8.4	169.1	0.4	169.9	169	169.2	173	176.4
201.7	9.0	199.0	9.3	200.2	3.8	200.2	199	200.4	205	199.1
321.5	12.9	318.1	0.5	319.6	8.5	320.0	318	318.6	322	318.5
347.9	11.9	345.6	13.2	346.7	9.4	346.6	346	346.4	350	342.5
417.7	9.9	414.4	10.6	415.8	5.2	416.2	415	415.7	421	432.0
476.6	10.9	475.2	14.9	474.8	11.3	474.9	475	n.o.	479	472.8
633.3	5.4	628.9	12.3	629.1	6.8	630.0	628	628.7	635	624.4
657.7	14.3	654.2	14.3	654.5	12.9	658.3	657	n.o.	663	655.8
767.2	13.8	765.5	15.5	765.5	13.3	766.7	763	763.9	772	767.0

## Data Availability

The data presented in this study are available on request from the corresponding author. The data are not publicly available due to the ongoing research.
